# A novel lipid metabolism-related lncRNA signature predictive of clinical prognosis in cervical cancer

**DOI:** 10.3389/fgene.2022.1001347

**Published:** 2022-10-17

**Authors:** Yanzhen Lu, Xiujun He, Xia Fang, Ningxia Chai, Fangfang Xu

**Affiliations:** Department of Gynaecology, The People’s Hospital of Beilun District, Beilun Branch Hospital of the First Affiliated Hospital of Medical School of Zhejiang University, Ningbo, China

**Keywords:** cervical cancer, lncRNA, lipid metabolism, signature, prognosis

## Abstract

**Background:** Cervical cancer (CC) is a serious threat to women populations worldwide. Lipid metabolism is believed to have modulating functions in cancer. Long non-coding RNAs (lncRNAs) are potential biomarkers for the different tumor prognosis. Our work aims at investigating the prognostic value of lipid metabolism-related lncRNAs in CC.

**Methods:** LncRNA expression profiling was conducted in 291 patients from The Cancer Genome Atlas (TCGA). Patient samples were randomly assigned to the training or testing set in a 3:2 ratio. A novel lipid metabolism-related five-lncRNA signature with prognostic value for CC was built through the univariate Cox regression, least absolute contraction and selection operator (LASSO) regression and multivariate Cox regression analyses, and was further evaluated by the Kaplan-Meier methods. Relevant analyses were also applied to identify the independent clinicopathological factors. GO and KEGG analyses were conducted to investigate the biological functions and molecular pathways. Immune infiltration analysis was included to probe the relationship between lncRNA signature and cancer cell microenvironment.

**Results:** The novel lipid metabolism-related five-lncRNA signature was confirmed to be predictive of overall survival (OS) in CC patients. Risk score, cancer stage, pregnancy, and BMI were validated as independent factors with prognostic value. GO and KEGG indicated that lipid metabolism participated in several tumor associated functions and pathways. Moreover, our results suggested that the five-lncRNA expression has potential link with tumor immune microenvironment.

**Conclusion:** In conclusion, we built an innovative prognostic risk signature based upon lipid metabolism-related lncRNAs. The five-lncRNA signature may be beneficial to provide novel potential therapeutic targets and improve personalized treatment strategies for CC patients in future clinical treatments.

## Introduction

Cervical cancer (CC) is a major cause of tumor-related death in female populations. In 2020, among the most common malignancies, CC ranks forth on a global scale (Sung et al., 2021). The situation was even worse in the developing countries owing to a poor screening coverage and low human papillomavirus (HPV) vaccination rate (Gakidou et al., 2008). Prominent risk factors for CC includes sexual activities at a young age, multiple sex partners, as well as immunosuppressive diseases which may increase vulnerability to HPV infection. Various treatment methods from chemotherapy, radiotherapy, immunotherapy to surgical interventions can be chosen according to different staging of patients (Tsikouras et al., 2016). And most importantly, prompt lesion detection and subsequent early CC diagnosis could help lead to less invasive therapeutic alternatives and improve total survival rate. Therefore, discovering new biomarkers and prognostic models are crucial for the prediction of patients’ outcomes and aid in early-stage interference.

Recently, there have been studies showing that abnormal lipid metabolism generally occurred in tumor cells at varied stages. Lipid metabolism disorder changes the microenvironment which has modulating functions on the growth, proliferation, invasion and metastasis of cancer cells (Ding et al., 2021; Peng et al., 2021). Nickels (2018) has demonstrated a strong correlation between increased lipid synthesis and accelerated metastatic processes in cancer. For instance, an increase in lipid synthesis has been detected in different types of tumor cells, which possibly accelerating their proliferation (Sethi et al., 2017; Wen et al., 2018). In addition, Zhang et al. (2020a) revealed the role of fatty acid metabolism reprogramming on CC lymph node metastasis (Shang et al., 2018), and in a subsequent study by Du et al., (2022) identified that fatty acid metabolism contributes to the poor prognosis of CC patients. This may provide us with possible lipid metabolism markers and potential therapeutic targets for CC. However, the lipid metabolism-related regulatory mechanism and key regulatory genes in CC has not been intensively studied. Long noncoding RNAs (lncRNAs), known as non-protein-coding RNA transcripts of over 200 nucleotides, drive many important cancer phenotypes by interacting with other macromolecules like DNA, protein, and RNA (Schmitt and Chang, 2016). They play a significant role in regulation of various cellular processes including cell growth and apoptosis (Renganathan and Felley-Bosco, 2017). Dysregulation of lncRNAs is commonly seen in tumor cells and are expressed differentially in different types of cancer. In the mean time, they presented to be associated with the patients’ clinical prognosis and survival (Choudhari et al., 2020). Nevertheless, the role and the prognostic value of lncRNAs in CC remains obscure at present.

The present study aimed at investigating the potential prognostic value of lipid-metabolism related lncRNAs in CC. To evaluate their association, lipid metabolism-related lncRNAs were screened for risk signature construction and a series of statistical analyses were conducted including the least absolute contraction and selection operator (LASSO) regression, univariate and multivariate Cox regression, Kaplan-Meier methods, functional enrichment, and immune infiltration analysis. In our study, we established a novel prognostic lipid metabolism-related lncRNAs signature for prediction of overall survival (OS) in CC. Our results shed new light on the prognostic value of lipid metabolism-related lncRNAs in CC and lay the foundation for potential cancer-targeted therapy.

## Materials and methods

### Patient data acquisition and processing

All patient information and their corresponding RNA-sequencing (RNA-Seq) data were obtained from The Cancer Genome Atlas (TCGA) database (https://portal.gdc.cancer.gov). A total of 860 lipid metabolism-related genes were downloaded from “Reactome metabolism of lipids and lipoproteins,” “Reactome phospholipid metabolism,” “Hallmark fatty acid metabolism” and “KEGG glycerophospholipid metabolism” in the Molecular Signature Database v5.1 (MSigDB) (Wu et al., 2019). These data were normalized by the “edgeR” package in R software. The demographic characteristics of all the included patients were demonstrated in Table 1.

**TABLE 1:** Baseline demographic characteristic.

Variables	Training set (*n* = 175)	Testing set (*n* = 116)
**Age, n (%)**		
≤60	140 (80.0%)	97 (83.6%)
>60	35 (20.0%)	19 (16.4%)
**Stage, n(%)**		
Stage I-II	134 (76.6%)	89 (76.7%)
Stage III-IV	41 (23.4%)	27 (23.3%)
**Grade, n(%)**		
G1-2	102 (58.3%)	57 (49.1%)
G3-4	73 (41.7%)	59 (50.9%)
**Number of pregnancies, n(%)**		
≤3	85 (48.6%)	57 (49.1%)
>3	70 (40.0%)	46 (39.7%)
unknown	20 (11.4%)	13 (11.2%)
**BMI, n(%)**		
≤24	48 (27.4%)	32 (27.6%)
>24	105 (60.0%)	68 (58.6%)
unknown	22 (12.6%)	16 (13.8%)
**Diagnoses, n(%)**		
Adenomas and Adenocarcinomas	19 (10.9%)	10 (8.62%)
Complex Epithelial Neoplasms	1 (0.57%)	2 (1.72%)
Cystic, Mucinous and Serous Neoplasms	10 (5.71%)	7 (6.03%)
Squamous Cell Neoplasms	145 (82.9%)	97 (83.6%)

### Prognostic long non-coding RNAs signature identification and subgroup survival analysis

Lipid metabolism-related gene (mRNA)—lncRNA coexpression was performed using the Pearson’s correlation analyses. Univariate Cox regression was conducted to further evaluate the lipid metabolism-related lncRNAs associated with patients’ survival. For building a lncRNA prognostic signature, all CC samples were randomly assigned to a training or testing set at a 3:2 ratio. Through the LASSO Cox regression, a prognostic risk signature was constructed. The established formula for calculating risk scores based on the expression levels of lipid metabolism-related lncRNAs was described as: Risk score = ∑ni = ∑Coefi × xi, where xi stands for the level of lncRNA i expression and Coefi for the regression coefficient. Determined by the median risk score, all patients were divided into high- and low-risk groups. Kaplan-Meier analysis were carried out to further estimate the differences in OS between the two groups in both training and testing cohorts. Receiver operating characteristic (ROC) analysis was used for assessing the accuracy of the risk model. Expression of lipid metabolism-related risk lncRNAs was visualized in a heatmap by the “pheatmap” R package.

To evaluate the correlations between survival and individual clinicopathologic factors, univariate and multivariate Cox regression analyses were carried out in both the training and testing sets. Survival analysis was conducted to evaluate the efficacy of the prognostic signature model by the Kaplan-Meier method.

### Functional analysis and immune cell infiltration

For biological function and signaling pathway analysis, Gene Ontology (GO) and the Kyoto Encyclopedia of Genes and Genomes (KEGG) pathways enrichment analyses were conducted in lipid metabolism-related mRNA expression with the clusterProfiler package in R. Besides, immune cell infiltration for 22 different genotypes were analyzed by CIBERSORT algorithm, and their differences between the two risk groups were evaluated.

### Statistical analysis

SPSS version 23.0 and R software version 4.0.2 were used for all statistical analyses. Differentially expressed lncRNAs were identified through the Benjamini–Hochberg method. Student *t*-test were performed for continuous variables and chi-squared (χ^2^) test for categorical variables. *p* < 0.05 was regarded as statistically significant.

## Results

### Identification of the prognostic lipid metabolism-related long non-coding RNAs in cervical cancer

**FIGURE 1:**
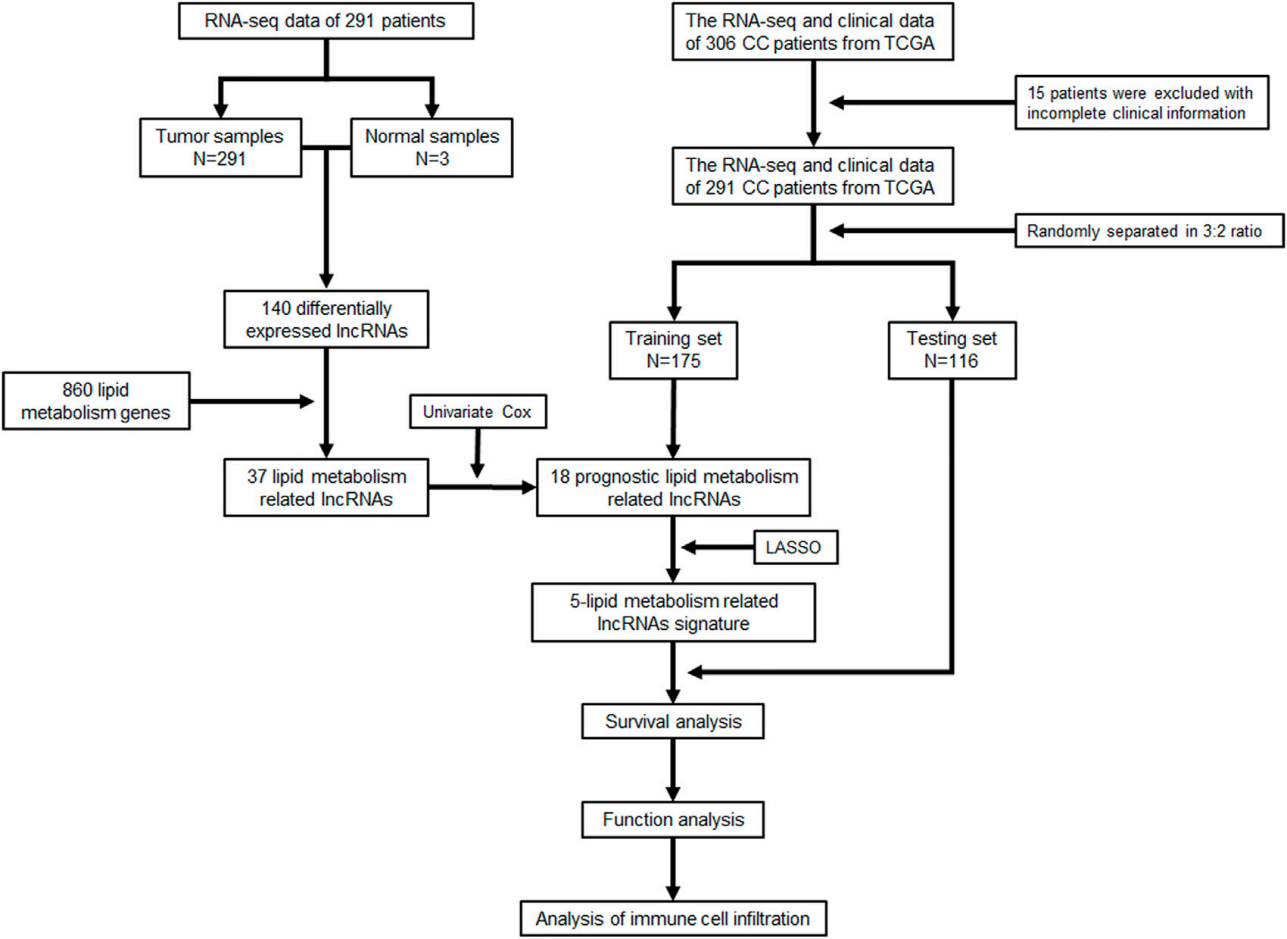
Flowchart of the study.

A total of 291 CC samples with RNA-seq data and clinical characteristics were obtained from the TCGA database for our study after excluding 15 patients for their incomplete clinical information. The workflow scheme for this study is illustrated in Figure 1.

**FIGURE 2:**
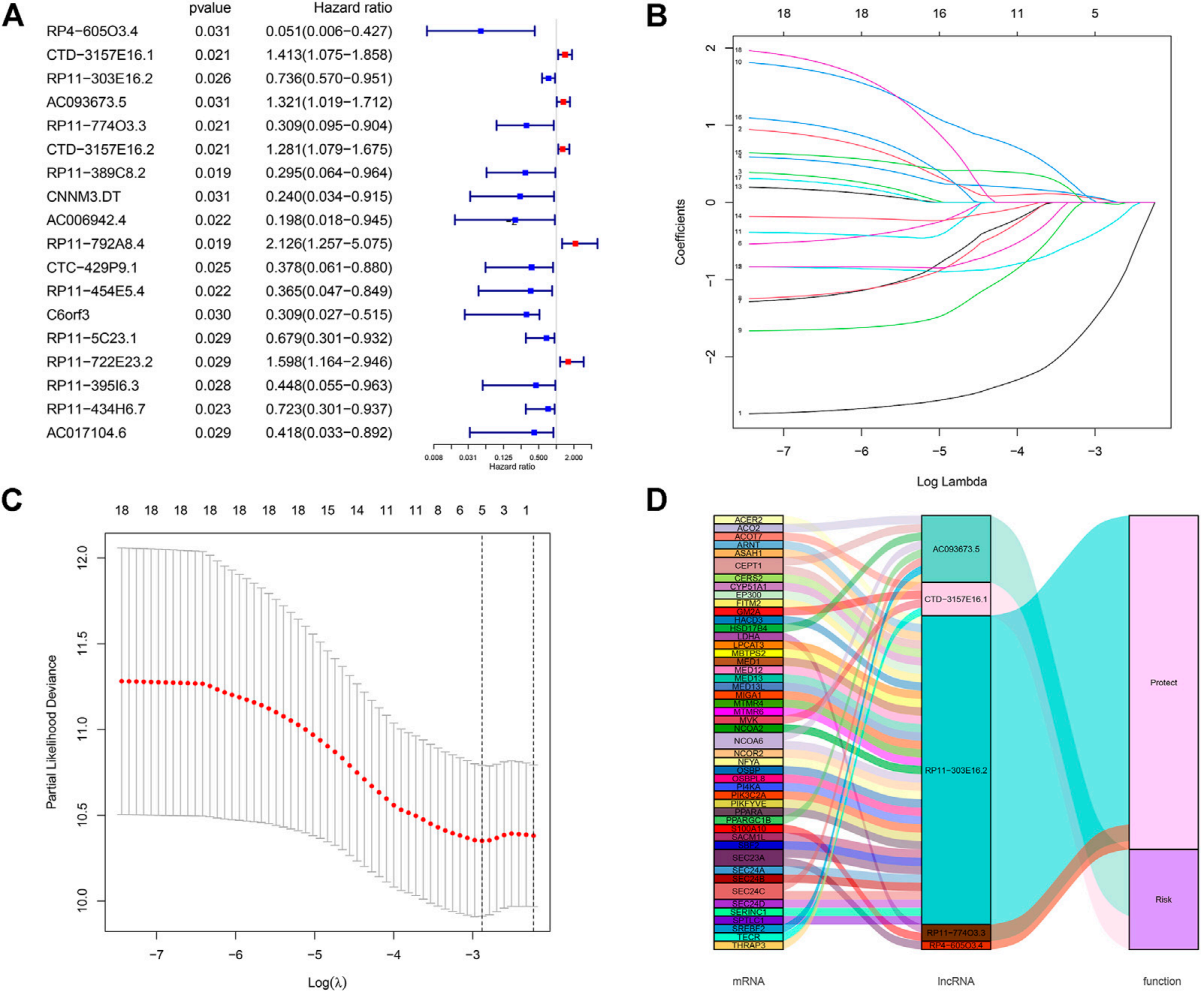
Screening of lipid metabolism-related lncRNAs with significant prognostic value in CC. **(A)** Forest plot illustrating the HR and *p*-value of 18 lipid metabolism-related lncRNAs by univariate Cox proportional hazards analysis; **(B,C)** LASSO Cox regression of the selected lncRNAs to calculate the minimum criteria **(B)** and coefficients **(C)**; **(D)** Alluvial map showing the relation between lipid metabolism-related mRNA and lncRNA, and their corresponding functions.

We performed differential expression analysis based on lncRNA expression data from tumor tissue samples and normal tissue samples of 291 CC patients in the TCGA database using the “edgeR” package in R software and obtained 140 differentially expressed lncRNAs. Thirty-seven lipid metabolism-related lncRNAs were identified by analyzing the coexpression between 140 differentially expressed lncRNAs with 860 lipid metabolism-related genes *via* the Pearson’s correlational analyses. Through the Cox proportional hazards analysis, eighteen lncRNAs significantly correlated with CC patients’ survival (*p* < 0.05) were identified, including 13 low-risk [hazard ratio (HR) < 1] and five high-risk (HR > 1) (Figure 2A).

### Construction and validation of a lipid metabolism-related long non-coding RNAs prognostic signature

All CC patients were distributed 3:2 to the training set (*n* = 175) or testing set (*n* = 116) at random. The above-mentioned 18 lncRNAs were used to construct a risk signature *via* the LASSO Cox regression analysis (Figures 2B,C). Five lipid metabolism-related lncRNAs were identified: CTD-157E16.1, AC093673.5, RP11-774O3.3, RP4-605O3.4, and RP11-303E16.2. Besides, an alluvial map was drawn to illustrate the relationship between lipid metabolism-related mRNA and lncRNA, and their corresponding functions (Figure 2D).

**FIGURE 3:**
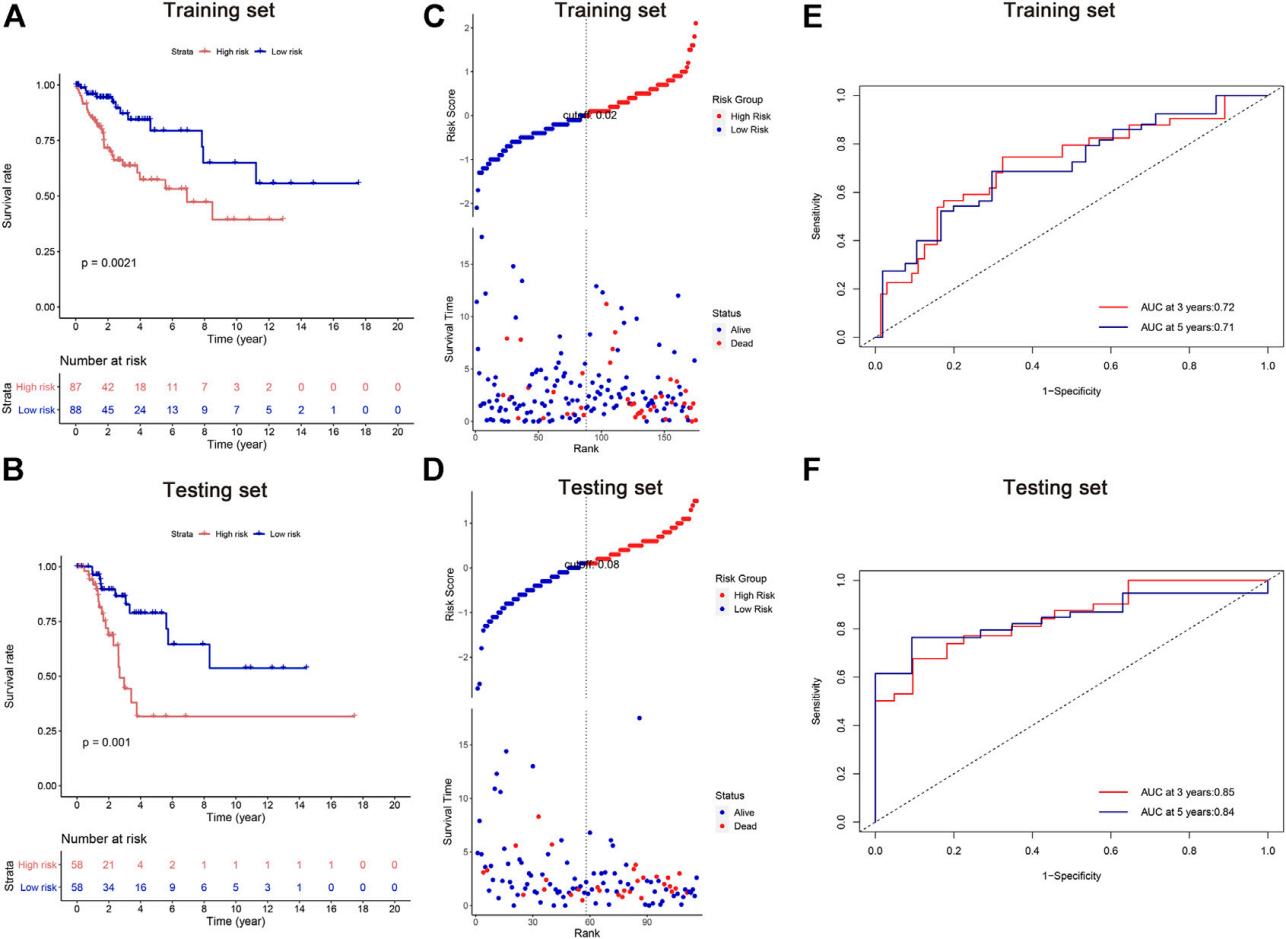
Survival analysis of cervical cancer patients in the training and testing sets. **(A,B)** Kaplan-Meier methods; **(C,D)** the distributions of risk scores, patient status, and survival time; **(E,F)** the receiver operating characteristic (ROC) analysis. The area under the ROCs (AUCs) were assessed for 3-year and 5-year survival.

According to their median risk score, patients in both training and testing cohorts were separated into high and low risk groups. Kaplan-Meier analysis revealed that the high-risk groups exhibited worse OS than their counterparts (Figures 3A,B). The distributions of risk scores, patient status, and survival time were evaluated, demonstrating that patients with high risk scores had a shorter survival time (Figures 3C,D). Moreover, the area under the ROCs (AUCs) were assessed for 3-year and 5-year survival. The results demonstrated promising predictive value of the five-lncRNA signature for CC patients (Figures 3E,F). All analyses were conducted in both training and testing sets, and similar results can be observed.

### Identification and validation of individual clinicopathological factors

**FIGURE 4:**
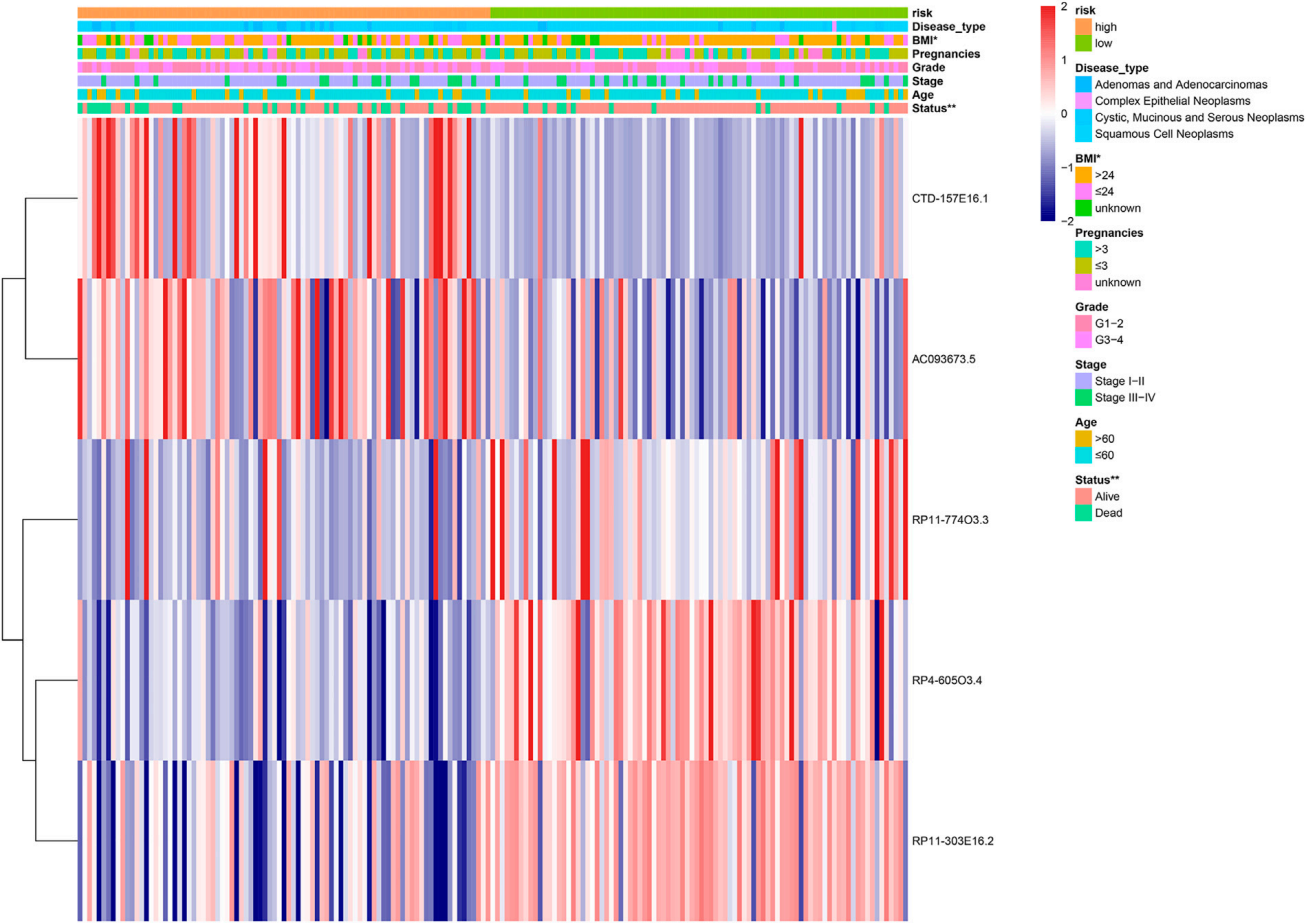
The heatmap showing the five-lncRNA expression in high and low cervical cancer (CC) patients. The CC-associated clinicopathological parameters was investigated between the high- and low-risk groups. *p < 0.05, **p < 0.01.

A heatmap was constructed to illustrate the associations between the five lipid metabolism-related risk lncRNAs and CC-related clinical characteristics. We found both CTD-157E16.1 and AC093673.5 as risky lncRNAs because their expression levels were inversely correlated to prognosis. On the contrary, RP11-774O3.3, RP4-605O3.4, and RP11-303E16.2 appeared as protective lncRNAs (Figure 3). Besides, we discovered that the risk score has significant relation with patient’s Body Mass Index (BMI) and status (Figure 4).

**FIGURE 5:**
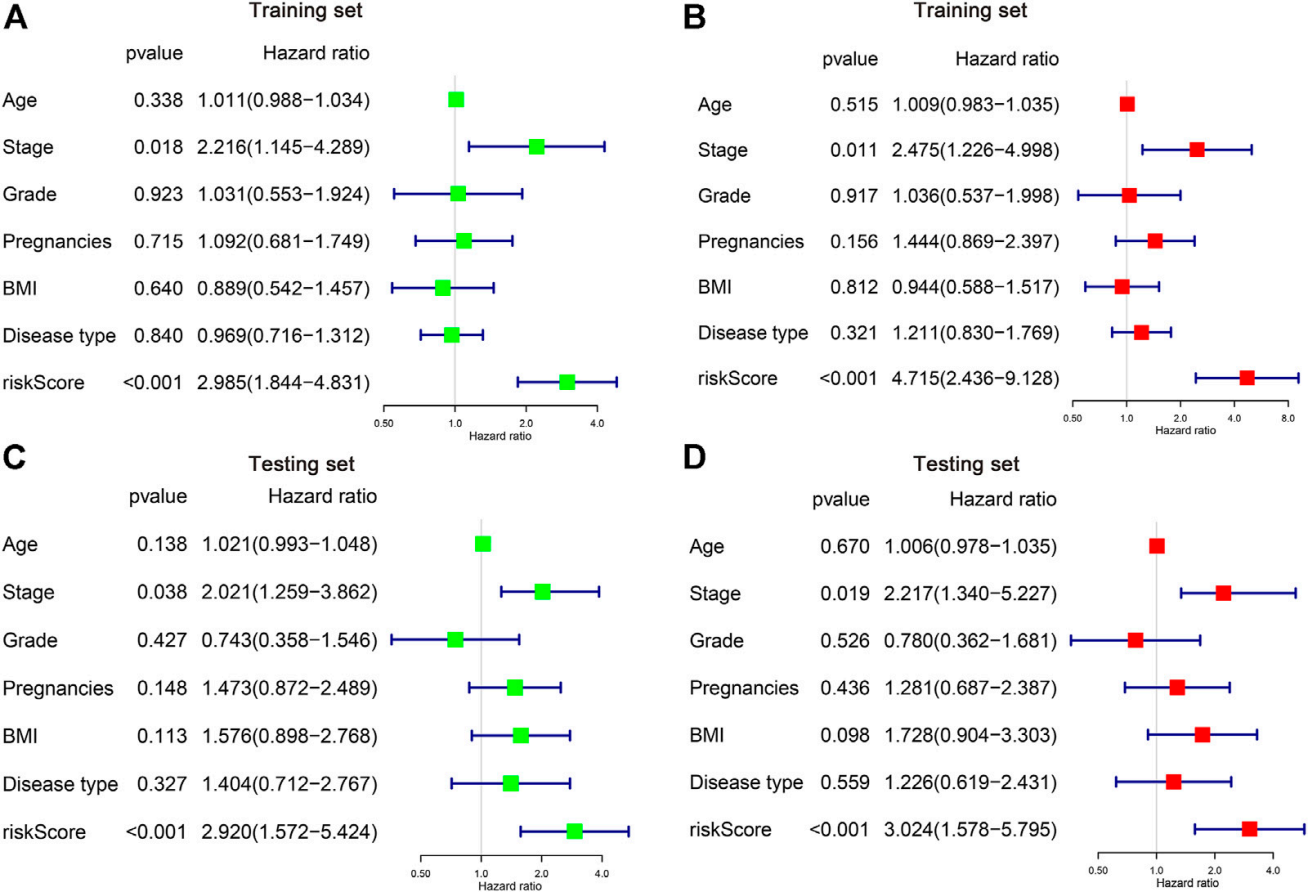
Identification of the independent risk factors in CC patients. **(A,B)** Univariate and multivariate Cox regression analysis of clinicopathological parameters were conducted in the training **(A,B)** and testing **(C,D)** cohorts.

We then applied both univariate and multivariate Cox regression models to investigate whether the risk score and other clinicopathological factors are possible independent parameters for CC prognosis. It is illustrated by the univariate Cox regression that the stage and the risk score was obviously associated with patient OS in both training and testing sets (*p* < 0.05) (Figures 5A,C). Similar results can be found in the multivariate analysis, showing the stage and the risk score was independent indicators of CC patient survival (Figures 5B,D).

**FIGURE 6:**
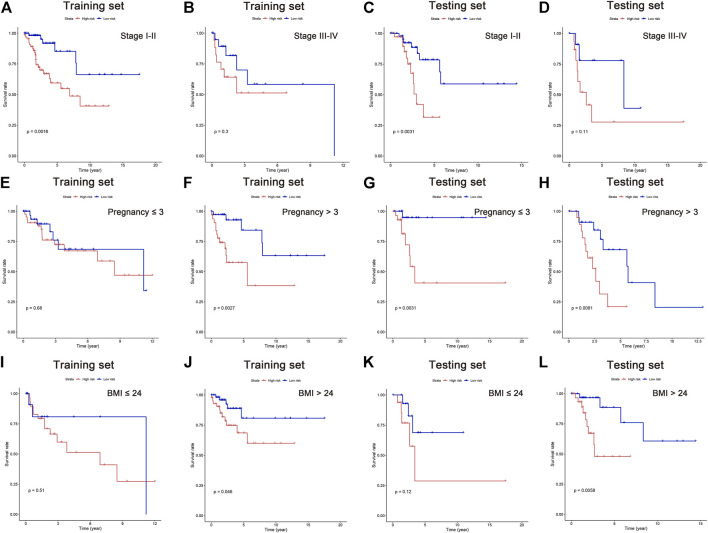
Survival analysis in subgroups of independent clinicopathological factors. Kaplan–Meier curves showing the prognostic value in subgroups of tumor stage **(A–D)**, pregnancy **(E–H)**, and BMI **(I–L)**.

It is suggested in some published literature that increased pregnancies and BMI could raise the risk for CC (Lima et al., 2006; Clark et al., 2016; Kim et al., 2021; Pasdar et al., 2021). Although no significant difference was found in pregnancy and BMI as independent risk factor in our above-mentioned analysis, their survival analyses were performed along with the clinical stage. In this study, all patients were classified into subgroups according to their cancer stage, pregnancy, and BMI. The results showed better OS in low-risk patients of stage I-II, pregnancy >3 times, and BMI >24 in the training set. Similar results can be observed in the testing set, showing better OS in stage I-II, pregnancy≦3 and >3 times, and BMI >24 in low-risk group (Figure 6A–L). These data indicated that the five lipid metabolism-related lncRNAs have a solid predictive value on CC prognosis.

### The potential role of lipid metabolism in cervical cancer and differences in immune cell infiltration

**FIGURE 7:**
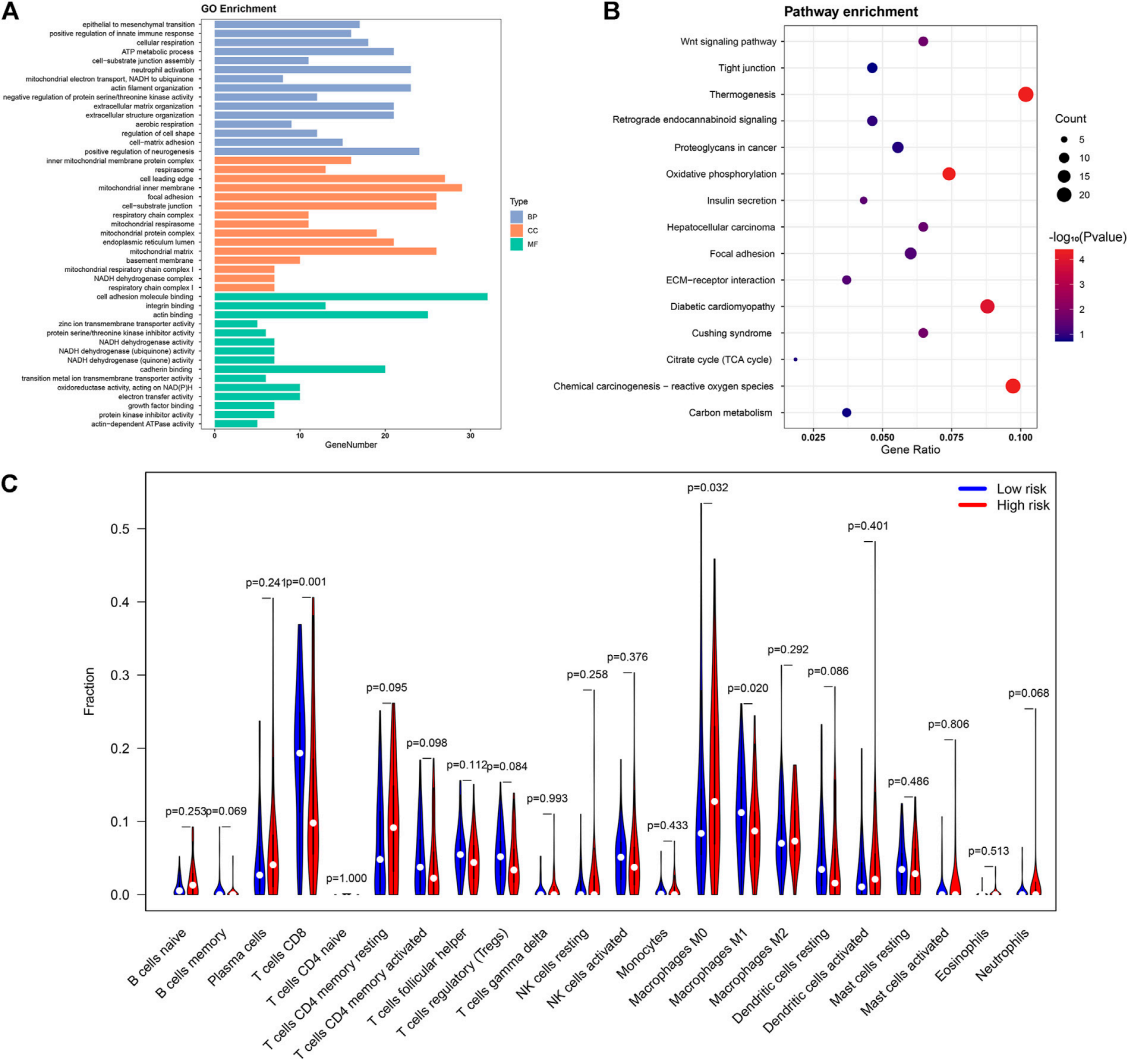
The potential role of lipid metabolism in CC and differences in immune cell infiltration. The functional analyses of lipid metabolism-related mRNAs using GO **(A)** and KEGG pathway analysis **(B)**; **(C)**The violin map showing differences in immune cell infiltration between low- and high-risk groups.

In the first part of the results, we identified 18 lipid metabolism related lncRNAs involved in CC patient survival. Based on the results of Pearson’s correlational analyses, we identified 462 lipid metabolism mRNAs associated with these 18 lncRNAs to probe into the biological functions and molecular pathways of the CC risk signature. Therefore, we carried out the GO annotation and KEGG analyses. Based on GO analyses, the biological functions of lipid metabolism mainly participated in cancer growth regulation, such as mitochondria biological process, cell adhesion, and actin binding (Figure 7A). In addition, the KEGG analyses revealed that these lipid metabolism mRNAs were enriched in some neoplasm-related pathways including carbon metabolism, ECM-receptor interaction, retrograde endocannabinoid signaling, and proteoglycans in cancer (Figure 7B).

To inquire into the correlation between immunocyte infiltration and patient risk score, differences in immune infiltration between the two risk groups were analyzed. Remarkably, compared with the low-risk group, CD8^+^ T cells and M1 macrophages showed significantly lower expression ratios in the high-risk group, whereas M0 macrophages were significantly higher in the high-risk group (Figure 7C). These results directly and indirectly revealed that our lipid metabolism related lncRNAs and mRNAs have potential links to the immune cell microenvironment in CC.

## Discussion

Accumulating evidence suggests that lipid metabolism related metabolic reprogramming has been considered as a hallmark of cancer (Shang et al., 2021), and lipid metabolism confers a tumor cell metastatic advantage (Nath and Chan, 2016; Pascual et al., 2017). To establish a convenient and efficient protocol to explore the lipometabolic status and predict clinical outcomes in CC patients, we established a novel lipid metabolism-related five-lncRNA signature for OS prediction. We found that patients with high risk had a significantly decreased three- and 5-year OS when compared with their low-risk counterparts. Risk score, tumor stage, pregnancy, and BMI were validated as independent prognostic factors by the univariate and multivariate Cox regression, as well as the survival analysis using Kaplan-Meier methods. In functional analysis, the newly developed lncRNAs mainly participated in cancer growth and progression and several tumor-associated pathways. Additionally, our results suggested that the five-lncRNA expression has potential link with tumor immunity by immune infiltration analysis.

The lipid metabolism related five-lncRNA signature was proposed for the first time based on tissue sample expression data and clinical information of CC patients. Compared with other proposed prediction models based, the five-lncRNA signature in this study more precisely illustrated the important role of lipid metabolism during CC progression. And is instructive for predicting the clinical outcomes of CC patients. The five-lncRNA signature proposal is promising to be applied in the future clinic for the immunohistochemical detection of tissue samples after CC surgery. By detecting the expression level of these five lncRNAs, clinicians can accurately judge the prognosis of CC patients and propose personalized clinical regimens. This will drive further development of targeted therapies in the future.

The proposed lipid metabolism-related risk signature in our study includes five lncRNAs: CTD-157E16.1, AC093673.5, RP11-774O3.3, RP4-605O3.4, and RP11-303E16.2. Xu et al. reported that AC093673.5 participated in allograft rejection and immunity, inducing chronic graft damage and allograft loss (Xu et al., 2020). The molecular mechanism might be associated chemokine-mediated signaling pathway, cellular response to interleukin and immunoglobulins. However, the functions of other four lncRNAs has not been investigated in published literature. Our study suggested that two lncRNAs (CTD-157E16.1 and AC093673.5) play a protective part in cancer cells development because their expression levels were inversely correlated to prognosis, whereas the other three act as the opposite role.

In our study, although no significant difference was found in pregnancy and BMI as independent risk factor by the univariate and multivariate Cox regression analysis, we performed the survival analysis for the two parameters along with cancer stage. We found that the stage, pregnancy and BMI were independent factors for CC prognosis. These results indicated that lipid metabolism-related lncRNA is a helpful and accurate tool in CC prognosis. In functional analysis, our results suggested that the lipid metabolism-related lncRNAs mainly participated in cancer progression and several tumor-associated pathways, such as carbon metabolism and proteoglycans in cancer. Published studies have demonstrated that carbon metabolism can alter the biological activities in CC for their role in DNA synthesis and repair (Tomita et al., 2013; Silva et al., 2020). Besides, various forms of proteoglycans have been indicated for potential inhibitive effect on cancer cell proliferation and invasiveness (Hirani et al., 2021; Yang et al., 2021).

Immune cells infiltration is believed to profoundly affect tumor growth and progression (Ferrall et al., 2021). Neoplasm-related macrophages can participate in the progression of CC *via* the STAT3‐miR‐223‐TGFBR3/HMGCS1 signaling pathway (Zhang et al., 2020b). Dendritic cells (DCs), which work as antigen-presenting cells (APC), have the potential to activate CD4^+^ and CD8^+^ T cells to induce tumor-specific response in CC (Lee et al., 2016). Neutrophils have been reported to be an important player in CC during different stages (Fomenko et al., 2018). Increased neutrophil populations can facilitate the progression and metastasis of CC. Our study illustrated that CD8^+^ T cells, M1 macrophages, and M0 macrophages has a strong correlation with risk score, indicating that these immunocytes possibly act as a crucial player in the formation and progression of CC. The result might potentiate novel immunotherapeutic drugs for the treatment of CC in future.

Nevertheless, several limitations exist in our study. First of all, our patient samples were relatively small in number and was downloaded from a single TCGA database. More data from other databases remains to be analyzed in future study. Secondly, our investigation is designed as retrospective with inevitable selection bias. Multicenter prospective researches are necessary for further confirmation of our results. In addition, in-depth experiments from molecular level should be carried out to further validate the function of our lipid metabolism-related lncRNAs and their role on cancer immunity during CC development.

As far as we are aware, the current study is the first to build a lipid metabolism-related lncRNA signature in CC. The five-lncRNA signature was validated in a series of analysis models, exhibiting its accuracy and reliability in CC prognosis. We hope more relevant investigations will be accomplished in future for further elucidating the roles and clinical applications of lipid metabolism-related lncRNAs in CC, as well as their underlying molecular mechanisms.

In conclusion, we constructed a novel prognostic signature based on lipid metabolism-related lncRNAs. The five-lncRNA signature may provide potential therapeutic targets for individualized CC treatment. Moreover, our results bring new insights into the role of lipid metabolism in tumor development and immune microenvironment of CC, which can be further investigated in future.

## Data Availability

The original contributions presented in the study are included in the article/supplementary material, further inquiries can be directed to the corresponding author.
